# Functional Heterogeneity in CD4^+^ T Cell Responses Against a Bacterial Pathogen

**DOI:** 10.3389/fimmu.2015.00621

**Published:** 2015-12-11

**Authors:** Ashley Viehmann Milam, Paul M. Allen

**Affiliations:** ^1^Department of Pathology and Immunology, Washington University School of Medicine, St. Louis, MO, USA

**Keywords:** CD4^+^ T cell, CD5, *Listeria monocytogenes*, immunomodulation, self-peptide, thymocytes, regulatory T cell

## Abstract

To investigate how CD4^+^ T cells function against a bacterial pathogen, we generated a *Listeria monocytogenes*-specific CD4^+^ T cell model. In this system, two TCRtg mouse lines, LLO56 and LLO118, recognize the same immunodominant epitope (LLO_190-205_) of *L. monocytogenes* and have identical *in vitro* responses. However, *in vivo* LLO56 and LLO118 display vastly different responses during both primary and secondary infection. LLO118 dominates in the primary response and in providing CD8 T cell help. LLO56 predominates in the secondary response. We have also shown that both specific [T cell receptor (TCR)-mediated] and non-specific stimuli (bypassing the TCR) elicit distinct responses from the two transgenics, leading us to conclude that the strength of self-pMHC signaling during development tightly dictates the cell’s future response in the periphery. Herein, we review our findings in this transfer system, focusing on the contribution of the immunomodulatory molecule CD5 and the importance of self-interaction in peripheral maintenance of the cell. We also discuss the manner in which individual TCR affinities to foreign and self-pMHC contribute to the outcome of an immune response; our assertion is that there exists a spectrum of possible T cell responses to recognition of cognate antigen during infection, adding immense diversity to the immune system’s response to pathogens.

## Introduction

The interaction of the T cell antigen receptor with its cognate antigen is essential for an adaptive immune response and involves the interaction between the T cell receptor (TCR) and peptide bound to an major histocompatibility molecule (MHC). Long before T cells meet cognate antigen in the periphery; however, they proceed through rigorous positive and negative selection in the thymus, according to the affinity of their rearranged TCR for self-peptide presented on self-MHC on thymic antigen-presenting cells (APCs).

The process of VDJ recombination and pairing of α and β chains allows for an astonishingly diverse panel of possible TCRs (1–3). There is a surprising level of redundancy inherent in the outcome of the combined processes of VDJ recombination and thymic selection, with a relatively limited number of unique TCRs able to recognize a broad spectrum of pathogen-derived antigens. There also exists the (non-mutually exclusive) phenomenon of multiple TCRs capable of recognizing the same peptide/MHC with similar affinities. What is the evolutionary advantage of such redundancy? It should be considered that heterogeneity in signaling responses following TCR engagement allows for the establishment of a full complement of key immune system players. This includes both effector and memory CD4^+^ and CD8^+^ T cells. Within the CD4^+^ T cell compartment, further specialization is accomplished via skewing of helper T (Th) cells, which produce key cytokines necessary for a full immune response and provide help to responding CD8^+^ T cells, and induction of regulatory T cells (Tregs) capable of preventing inflammatory damage during an otherwise overzealous immune response.

So, how do individual TCR affinities to foreign and self-pMHC contribute to the outcome of an immune response? To better address this question, we have designed a T cell system involving transfer of two congenically marked TCR-transgenic T cells (LLO56 and LLO118), both recognizing the same *Listeria monocytogenes*-derived epitope. The T cells are transferred into normal B6 recipient mice, which are in turn infected with *L. monocytogenes*. *In vitro*, these two naïve T cells have very similar antigen responses to both peptide and intact *Listeria*. Not until they are activated *in vivo* can one appreciate the vastly different potential of the two. One, LLO118, responds robustly during primary infection, only to make a poor showing during a secondary response, where LLO56 dominates. Unlike LLO118, LLO56 is highly apoptotic following the primary immune response. Differential expression of CD5 associated with the two TCRs helps explain their differing *in vivo* phenotypes. Intriguingly, these cells diverge in their response to both antigen-specific stimuli and non-specific stimuli, which bypasses TCR signaling altogether, indicating that self-peptide-dictated imprinting during thymic selection and maintenance in the periphery can critically affect all aspects of behavior throughout the lifespan of a T cell.

## Derivation and Characterization of Listeriolysin O-Specific TCR-Transgenic Mice

The T cell receptors of the LLO56 and LLO118 mouse lines were originally cloned from a panel of T cell hyrbidomas generated from B6 mice infected with *L. monocytogenes*. The two TCRs recognize the same immunodominant epitope of listeriolysin O (LLO_190-205_/I-A^b^), and both express Vα2 and Vβ2. The TCR sequences of these cells are nearly identical, differing by only 15 amino acids in the complementary determining regions (CDRs). Flow cytometry-based analysis of the two transgenics shows that LLO56 and LLO118 have similar naïve phenotypes, the only notable exception being their expression of CD5, a negative regulator of T cell activation (4). CD5 surface expression correlates with TCR self-reactivity, as CD5 levels are determined during thymic T cell development according to the strength of signal perceived via TCR–self-pMHC engagement during thymic T cell development (5). The LLO56 mouse expresses significantly higher levels of CD5 on mature thymocytes, splenocytes, and peripheral lymph node (LN) CD4^+^ T cells. LLO56 and LLO118 have similar functional affinities for LLO_190-205_/I-A^b^, as measured by their *in vitro* proliferative response to peptide stimulation or stimulation with *L. monocytogenes*-infected splenocytes. Surface plasmon resonance (SPR) analysis of the soluble LLO118 and LLO56 TCR interaction with LLO_190-205_/I-A^b^ revealed that they have the same overall affinity (*K*_D_).

In our standard transfer system, 3 × 10^3^ congenically marked, CD4^+^ T cell-enriched bead sorted cells from LLO56 *Rag1*^−/−^ and LLO118 *Rag1*^−/−^ mice are co-transferred into B6 recipient mice via intravenous (IV) retro orbital injection on day 0. On day 1, recipient mice are infected (IV) with 10^3^ CFU *L. monocytogenes* (1043S). Mice are then sacrificed at day 7 to interrogate the primary immune response. To analyze the recall response, mice are re-infected with 10^5^ CFU *L. monocytogenes* on day 35 and then sacrificed on day 39.

LLO56 and LLO118 respond distinctly at both the primary and secondary time points. Annexin V staining reveals that LLO56 are highly apoptotic by day 7 (4). LLO118 are recovered from the spleens of recipient mice at a ratio of ~5:1 the number of LLO56 recovered. This ratio holds even as the numbers of injected cells are increased 10-fold or if LLO56 and LLO118 cells are transferred into separate mice. Therefore, the difference in response is not due to MHC-antigen competition nor a limited proliferative niche, but rather inherent differences in the capacity of the two cell types to respond to infection.

Interestingly, the secondary response in our transfer model is dominated by LLO56 cells, which outstrip LLO118 cells at a ratio of ~10:1. We hypothesize that this is due at least in portion to the massive downregulation of TCR levels observed on LLO118 cells recovered after a primary infection (4). Starting at day 8, this difference in TCR mean fluorescence intensity (MFI) in the two cells is on the order of 1–1.5 logs, and it has been shown that TCR downregulation can greatly reduce the proliferative ability of T cells (6).

We also compared the ability of LLO56 and LLO118 to provide CD4^+^ T cell help for a CD8^+^ T cell response in the context of a primary *L. monocytogenes* infection. Using a transfer model similar to that described above, with the addition of the *L. monocytogenes*-OVA system, we found equal numbers of OVA-specific CD8^+^ T cells (as measured by tetramer staining) in mice co-injected with either LLO56 or LLO118 CD4^+^ T cells during a primary response. However, significantly more OVA-specific CD8^+^ T cells were found after a recall response in mice co-transferred with LLO118 CD4^+^ T cells. Thus, the more robust LLO118 primary response correlates with a better CD8^+^ T cell response after secondary infection (4).

Further insight into the proliferative and memory-forming capacities of these cells was achieved using analysis of three different mathematical models that considered the differentiation of naïve cells first into effector and then memory cells. While the three ­models reach no consensus on a difference in the rate of ­memory cell formation in LLO56 and LLO118 cells, LLO56 memory T cells are predicted to have a half-life nearly three times longer than LLO118 memory T cells $\left(t_{\text{1}/\text{2}}^{\text{118}}\approx \text{4}.\text{3}-\text{5d vs}.\;t_{\text{1}/\text{2}}^{\text{56}}\approx \text{11}.\text{5}-\text{13}.\text{9 days}\right)$. This predicted ­difference in memory maintenance would explain the better performance of LLO56 during a recall response (7).

## T Cell Responsiveness to Specific and Non-Specific Stimuli is Set During Thymic Development and Maintained in the Periphery

*In vitro* analysis of LLO56 and LLO118 revealed inherent differences in the manner in which the two TCRs to respond to stimulus, be it antigen specific (that is, perceived through the TCR) or non-specific. When LLO56 and LLO118 were stimulated *in vitro* with either their cognate antigen or a combination of anti-CD3/anti-CD28, both cells upregulated CD25 and CD69, and produced IL-2 (8). However, LLO56 produces nearly twice as much IL-2 as LLO118 at higher peptide (or anti-CD3/28) concentrations. However, LLO56 also produced more IL-2 in response to treatment with PMA plus ionomycin, which stimulate signaling downstream of the TCR. Further examination of the pathways activated by PMA plus ionomycin also showed increased phosphorylated Erk (pErk) in LLO56 in response to non-specific stimuli, as well as higher basal p21 (increased basal levels of phosphorylated TCRζ). These findings indicate that naïve LLO56 and LLO118 emerge from the thymus distinct in their capacities to respond to both cognate antigen and non-specific stimulation. While their *in vitro* affinities for LLO_190-205_/I-A^b^ are similar, their avidities (the strength of signal actually perceived by the cell via the TCR) clearly differ.

To better understand the behavioral divergence of these two transgenics, we interrogated thymocytes from LLO56 and LLO118 mice at each stage of development. Although the absolute cellularity of the thymus in the LLO118 mouse is greater, we found that the frequency of CD4^+^ single-positive (SP) thymocytes to be greater in the LLO56 mouse. This suggests that selection of CD4^+^CD8^+^ double-positive (DP) thymocytes is more efficient in the context of the LLO56 TCR. While pre-selection DP thymocytes from both mice are refractory to PMA plus ionomycin stimulation, we found a population of CD4^+^SP thymocytes in both LLO56 and LLO118 mice producing IL-2; this population is significantly greater in the LLO56 mice. Likewise, phospho-ERK staining is higher in CD4^+^SP LLO56 thymocytes. We also observed greater Annexin V and 7-aminoactinomycin-D (7-AAD) staining in post-selection LLO56 thymocytes than in LLO118 thymocytes, indicating that increased cell death accompanies increased basal ERK phosphorylation, according to TCR self-reactivity and as established in developing thymocytes (8).

Since we had documented the importance of self-pMHC education in the earliest development of LLO56 and LLO118, we sought to determine whether continued tonic self-peptide–MHC signaling was necessary for their peripheral maintenance and responsiveness. To accomplish this, LLO56 splenocytes were transferred to H-2M deficient mice, which fail to present a normal range of processed peptides due to the fact that nearly all MHC class II molecules are occupied by class II invariant chain-associated peptide (CLIP). LLO56 cells were then recovered and purified, and their responsiveness to non-specific stimuli tested. Unlike their counterparts transferred into wild-type B6 mice, LLO56 cells transferred into H-2M-deficient mice lost sensitivity to stimulation via PMA plus ionomycin, as measured by IL-2 production and ERK phosphorylation and were similar to the LLO118 cells. Likewise, we also observed reduced ERK phosphorylation in LLO56 cells transferred into MHC class II-deficient mice (8). These behavioral changes were noticeable as early as 24 h post-transfer. These findings indicated that continued TCR–self-peptide–MHC ligation is critical for the preservation of T cell responsiveness.

## CD5 and the Dynamics of LLO TCRtg T Cell Responses

CD5 belongs to group B of the scavenger-receptor cysteine-rich (SRCR) superfamily; the extracellular portion of CD5 consists of three SRCR repeats. In mice, the *Cd5* gene encodes a 67-kDa monomeric membrane-spanning glycoprotein expressed on thymocytes, mature CD4^+^ and CD8^+^ T cells, as well as peritoneal B-1 B cells and subsets of dendritic cells (9–12). CD5 species-specific homophilic binding can lead to productive engagement; other CD5 ligands have been reported, but none have been independently verified (13). The molecular mass and expression pattern of CD5 in humans is similar to that in mice. Indeed, the high level of conservation of the Cd5 gene throughout mammalian and avian species suggests an ancient and critical role for CD5 in the immune system (14–17). The finding that a naturally occurring soluble form of CD5 is a pattern-recognition receptor (PRR), which is capable of recognizing fungal β-glucan (but not components of bacterial cell wall), reinforces the evolutionary significance of this molecule (18).

The role of CD5 as an immunomodulatory cell surface molecule has been appreciated since the publication of the *Cd5*-knockout mouse (15, 19). CD5 is capable of regulating signaling via both the TCR and the B cell receptor (BCR). In the absence of CD5, thymocytes are hyperresponsive to antigen stimulation (as measured by Ca^2+^ signaling) and peritoneal B-1 cells, levels of which are elevated in certain autoimmune diseases, become resistant to apoptosis and instead enter the cell cycle. However, CD5-knockout mice do not develop significant overt immunity (19, 20). In addition to its expression on peripheral effector T cells, CD5 is also expressed on CD4^+^ Tregs. While CD5 is dispensable for thymic Treg development (21), peripheral Treg induction is impaired in its absence (22).

The immunomodulatory nature of CD5 is independent of its extracellular domains, but an intact cytoplasmic domain is required for its inhibitory function (23, 24). CD5 localizes to the immune synapse at the onset of TCR signaling (25). The intracellular domain of CD5 contains four potential tyrosine phosphorylation sites, including an immunoreceptor tyrosine-based activation motif (ITAM), immunoreceptor tyrosine-based inhibition motif (ITIM), and several potential serine/threonine phosphorylation sites (26). In this way, CD5 is able to recruit both negative and positive regulators of B and T cell signaling (27). CD5 is tyrosine phosphorylated upon TCR engagement, and coprecipitaiton studies have demonstrated that CD5 associates with CD2 and CD4 or CD8, as well as with TCR ζ/Zap70, Lck, Fyn, SHP-1, and CK2. SHP-1 binding to the intracellular ITIM motif mediates the immunomodulatory behavior of CD5 (28–33).

Considerable work has been done to show the precision with which CD5 expression is regulated during thymic development. CD5 expression on pre-selection double-negative (DN) thymocytes is minimal; these developing thymocytes require pre-TCR engagement for upregulation of CD5 to be observed (5). CD5 levels increase approximately sixfold on DP thymocytes and reach maximal levels on post-selection CD4^+^ and CD8^+^SP thymocytes, where expression is finely tuned according to TCR levels and the overall perceived strength of signal during selection (5). CD5 levels are then maintained as thymocytes egress from the thymus and join peripheral circulation. These findings have been extended to commonly used TCR-transgenic mouse models AND (CD4^+^ TCR transgenic recognizing moth cytochrome *c*), H-Y (CD4^+^ TCR transgenic recognizing the male Y antigen), P14 (CD8^+^ TCR transgenic recognizing LCMV gp33-41), and DO10 (CD8^+^ TCR transgenic recognizing OVA). Examination of thymocytes from these mice revealed higher CD5 levels on AND than on H-Y, and higher CD5 levels on P14 than on DO10. Likewise, larger CD4^+^SP thymocyte populations are observed in AND relative to H-Y, and larger CD8^+^SP populations are observed in P14, relative to DO-10 (5). A study using the DO10 mouse in the context of both H-2^d^ and H-2b-mediated antigen presentation echoed these findings, as did another that extended findings on the relationship between CD5 expression and self-reactivity to a larger panel of TCR transgenics (23, 34). The CD5 levels studied are maintained on peripheral lymphocytes, reinforcing the finding that CD5 expression is carefully tuned during thymic selection according to TCR–self-peptide–MHC signal intensity, and sustained during the life of the cell as long as MHC presentation of self-peptide is accessible in the periphery.

Is has been debated whether CD5 directly influences T cell responses, or whether it is simply a marker of TCR–self-peptide–MHC avidity, established during thymic selection. To explore this issue in our system, we crossed LLO56 TCR-transgenic mice onto a CD5-deficient background (CD5^−/−^). CD4^+^SP thymocytes from LLO56/CD5^−/−^ mice express higher levels of CD69 than their wild-type counterparts, indicating that in the absence of CD5 these thymocytes perceive a stronger signal during selection. These cells also produce more IL-2 and exhibit greater ERK phosphorylation in response to non-specific stimulation, in the absence of CD5 expression (8). Surprisingly, the absence of CD5 on the LLO56 background (LLO56/CD5^−/−^) does not change its apoptotic phenotype at day 7 post-infection, in our transfer system (unpublished observations). CD5 can be viewed as a “surrogate marker” of the TCR signal experienced during thymic education, and it also appears that CD5 (as a negative regulator) wields some influence on the overall responsiveness of the T cell post-selection. However, at least in the context of our TCR-transgenic system, loss of CD5 inhibition is not sufficient to “rescue” the phenotype of LLO56 at day 7. Clearly, further interrogation of the role of CD5 during an ongoing immune response is necessary.

These observations also bring up relevant findings regarding CD5 expression and the induction of peripheral Tregs. Henderson and colleagues recently reported that peripheral induction of CD4^+^ Tregs is decreased in CD5^lo^ cells and cells from CD5^−/−^ mice. They found that low levels of effector cytokines produced by bystander lymphocytes inhibited Treg conversion in these mice. In CD5 intact mice, on the other hand, CD5^hi^ cells are able to mitigate this inhibition of Treg induction via blockade of mammalian target of rapamycin (mTOR) signaling (22). This study reinforces the findings of Martin and colleagues, who showed that Ly6C^lo^ and Ly6C^hi^ naïve CD4^+^ T cells have intrinsic differences when it comes to their abilities to differentiate into Tregs in the periphery. The authors demonstrated that Ly6C expression was tuned in the periphery according to TCR-based self-recognition, and that more self-reactive cells exhibited lower surface levels of Ly6C. In turn, they showed that Ly6C^lo^ cells had significantly higher levels of CD5 and were more likely to differentiate into Tregs in the periphery (35). These findings, along with the observation that minimal doses of strong agonist peptides can efficiently induce Treg differentiation in the periphery, suggests a dual role for CD5 (35, 36). Clearly, CD5 serves as a marker of self-reactivity on both thymocytes and lymphocytes. Strong CD5 expression “tags” these self-reactive naïve cells as those most likely to become activated, with great specificity and intensity, during an immune response. At the same time, CD5 also serves as an “insurance policy,” helping keep these potentially destructive cells from initiating autoimmunity and instead dedicating them, via induction of Treg programming, to the protection of the organism in question from a potentially devastating inflammatory episode.

Although only the LLO system is reviewed herein, it stands to reason that these findings can be extended to other models of infectious disease. Presumably, during an infection, there will be a large number of foreign antigens presented to T cells, which in turn will have been selected on a wide variety of self-antigens during thymic development and will therefore express varying levels of CD5. By extension of our findings, we would predict that within this T cell compartment are cells that will respond robustly (in terms of effector function, B cell help, T cell help, and/or regulatory function) and cells that will respond to a lesser degree, giving breadth to the immune response as a whole.

## Summary

Using our LLO TCR-transgenic transfer system, we have demonstrated the importance of heterogeneous responses by different CD4^+^ T cells following TCR engagement. The CD4^+^ TCR-transgenic LLO56, bearing high levels of CD5 reflective of strong sensing of self-pMHC during thymic development, responds poorly during a primary response to cognate antigen. LLO118, on the other hand, has a robust proliferative response to the same cognate antigen and, at the same time, provides help during the shaping of the CD8^+^ T cell response. We see the responses of LLO56 and LLO118 reverse during secondary infection, where LLO56 dominates the recall response.

**Figure 1:**
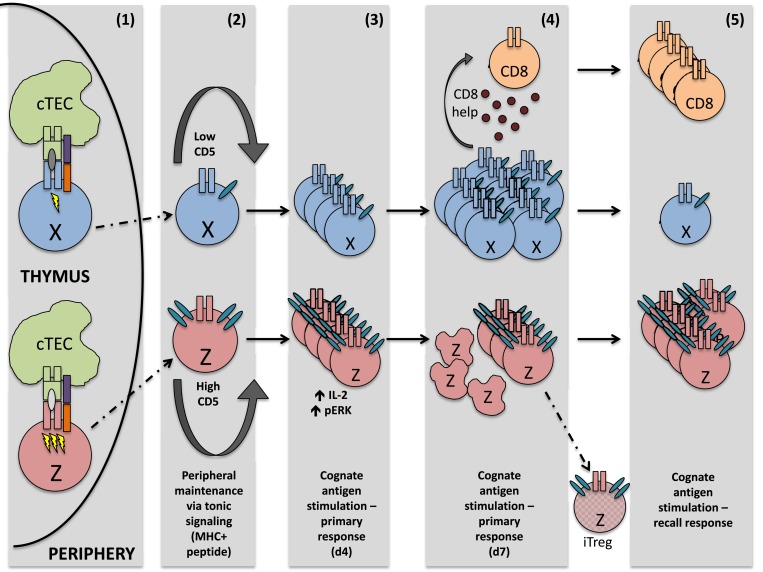
**After interaction with self-peptide on thymic APC, both cells perceive a signal strong enough to guide them safely through positive selection**. However, the signal sensed by cell Z is quantitatively or qualitatively stronger, resulting in greater CD5 expression on the mature thymocyte Z, relative to mature thymocyte X. Upon egress from the thymus, these differing CD5 levels are sustained. In the periphery, both naïve T cells are maintained via tonic signaling (i.e., periodic TCR recognition of self-pMHC). Upon initial TCR recognition of the same peptide, cell Z exhibits increased levels of phosphorylated ERK and produces higher amounts of IL-2, relative to cell X. However, by the peak of the immune response, cell X is highly proliferative and capable of generating CD8^+^ T cell help, whereas cell Z is highly apoptotic and a poor generator of CD8^+^ T cell help. Some cell Z clones, however, may differentiate into regulatory T cells, due at least in part to their high expression of CD5. During a recall response, memory cell Z now proliferates strongly in response to the same cognate antigen sensed during the primary immune response, while proliferation of cell X is negligible.

We have highlighted only these two distinct cell fates using our system; however, there is ostensibly a whole “spectrum” of possible CD4^+^ T cell responses to recognition of cognate antigen during infection, adding necessary diversity to the immune system’s response to pathogens. Two such outcomes, along the spectrum of possible CD4^+^ T cell responses, are mapped in Figure 1. Importantly, this spectrum of fates includes activation of cells that respond robustly to acute insults, cells that preferentially become memory cells, cells specialized in providing CD8^+^ T cell help, and cells more easily drawn into a Treg fate, just to name a few. These properties are not necessarily mutually exclusive and are determined in large part by recognition of self-peptide during thymic selection. A strong case has also been made for the role of cross-reactivity (that is, the ability of one TCR to recognize multiple pMHC) during thymic selection. It should also be considered that increased CD5 expression on LLO56 could be due, at least in part, to an ability of LLO56 to cross-react more strongly with self-antigens in the thymus (37–40).

## Author Contributions

AM and PA co-wrote and edited this article.

## Conflict of Interest Statement

The authors declare that the research was conducted in the absence of any commercial or financial relationships that could be construed as a potential conflict of interest.
